# Supplementary material for: Flubendazole Enhances the Inhibitory Effect of Paclitaxel via HIF1α/PI3K/AKT Signaling Pathways in Breast Cancer
Source: Int J Mol Sci. 2023 Oct 12;24(20):15121. doi: 10.3390/ijms242015121 (PMC10606573; doi:10.3390/ijms242015121)
Supplement: Supplementary file 1 [file ijms-24-15121-s001.zip › ijms-2576452-supplementary.pdf]

Supplementary Material

Flubendazole Enhances the Inhibitory Effect of Paclitaxel via HIF1α/PI3K/AKT Signaling Pathways in Breast Cancer

Yuxin Zhou [1], Minru Liao [1], Zixiang Li [2], Jing Ye [1], Lifeng Wu [1], Yi Mou [1], Leilei Fu [2]* and Yongqi Zhen [1]*

[1] Department of Biotherapy, Cancer Center and State Key Laboratory of Biotherapy, West China Hospital, Sichuan University, Chengdu 610041, China; charlotte_zyx@outlook.com (Y.Z.); 15680965456@163.com (M.L.)

[2] Sichuan Engineering Research Center for Biomimetic Synthesis of Natural Drugs, School of Life Science and Engineering, Southwest Jiaotong University, Chengdu, 610031, China; li69010@163.com (Z.L.); scuyj0222@163.com (J.Y.); 18482273923@163.com (L.W.); skayhy@163.com (Y.M.)

* Correspondence: leilei_fu@swjtu.edu.cn (L.F.); zhenyongqi@scu.edu.cn (Y.Z.).

Supplementary Figure S1

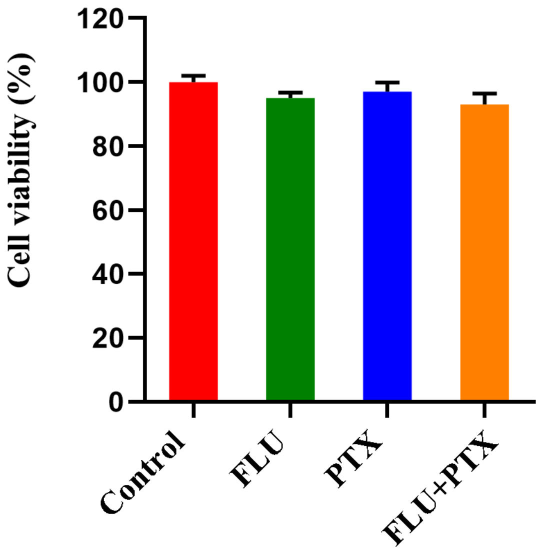

Figure S1. Cell viability of flubendazole and paclitaxel on normal breast cells (MCF-10A). Cell viabilities were measured by MTT assay. Data are expressed as mean ± SEM. All data were representative of at least three independent experiments. Statistical significance compared with respective control groups.
